# Notch Signaling Pathway and Endocrine Resistance in Breast Cancer

**DOI:** 10.3389/fphar.2020.00924

**Published:** 2020-06-19

**Authors:** Jing-Wen Bai, Min Wei, Ji-Wei Li, Guo-Jun Zhang

**Affiliations:** ^1^Department of Oncology, Xiang’an Hospital of Xiamen University, School of Medicine, Xiamen University, Xiamen, China; ^2^Cancer Research Center, School of Medicine, Xiamen University, Xiamen, China; ^3^Department of Breast and Thyroid Surgery, Xiang’an Hospital of Xiamen University, School of Medicine, Xiamen University, Xiamen, China; ^4^Clinical Central Research Core, Xiang’an Hospital of Xiamen University, Xiamen, China

**Keywords:** breast cancer, Notch signaling pathway, endocrine resistance, estrogen receptor, anti-estrogen therapy

## Abstract

Nearly 70% of breast cancers express the estrogen receptor (ER) and are hormone-dependent for cell proliferation and survival. Anti-estrogen therapies with aromatase inhibitors (AIs), selective estrogen receptor modulators (SERMs) or selective estrogen receptor down regulators (SERDs) are the standard endocrine therapy approach for ER positive breast cancer patients. However, about 30% of patients receiving endocrine therapy will progress during the therapy or become endocrine resistance eventually. The intrinsic or acquired endocrine resistance has become a major obstacle for endocrine therapy. The mechanism of endocrine resistance is very complicated and recently emerging evidence indicates dysregulation of Notch signaling pathway contributes to endocrine resistance in breast cancer patients. The potential mechanisms include regulation of ER, promotion of cancer stem cell (CSC) phenotype and mesenchymal cell ratio, alteration of the local tumor microenvironment and cell cycle. This review will summarize the latest progress on the investigation of Notch signaling pathway in breast cancer endocrine resistance.

## Introduction

Breast cancer has become the leading common cancer and the second largest cause of death among women worldwide (Siegel et al., 2020). A majority of breast cancer shows ER expression (Yip and Rhodes, 2014) and anti-estrogen therapy is considered as the most effective treatment for them. Current anti-estrogen drugs include SERMs (i.e., tamoxifen), AIs (i.e., letrozole and anastrozole), and SERDs (i.e., Faslodex/Fulvestrant). It has been considered a revolutionized progress in endocrine therapies which significantly decreases cancer-related mortality and improves the survival rate (Tremont et al., 2017). But about 30% patients treated with endocrine therapy will develop recurrence even though initially respond well (D'Souza et al., 2018). Thus the resistance has been believed as a pivotal obstacle leading to breast cancer treatment failure.

The endocrine mechanisms in breast cancer are complex and multiple with diverse molecules and pathways involved. 1) Direct or indirect ER related signaling pathway: As ERα has been proven to be the main target in endocrine therapy, changes in ERα expression or function including ERα loss or ESR1 mutations or epigenetic modification all contribute to endocrine independence (Toy et al., 2013; Gelsomino et al., 2016; Tecalco-Cruz and Ramirez-Jarquin, 2018; Fontes-Sousa et al., 2019). 2) Non-ER related signaling pathways, such as promotion of stemness of cancer cells and EMT, dysregulation of cell cycle, crosstalk with cells tyrosine kinase growth factor signaling pathways, influence of tumor microenvironment and drug metabolism also act crucial parts in endocrine resistance (Rani et al., 2019).

Both experimental studies and clinical observations suggested that the aberrant activation of Notch signaling pathway was very common in breast cancer and it was depicted in most of regulating pathway related to endocrine resistance (Acar et al., 2016).

In this review, we will sum up the latest development aiming at the role of Notch signaling pathway and discuss the complicated evidence underlie its impact on endocrine resistance. The potential for Notch correlated cancer therapy is also highlighted.

## The Notch Signaling Pathway

Notch signaling pathway is highly conserved in eukaryotes which involves two kinds of adjacent cells, signal sending and receiving cells (Wilson and Radtke, 2006; Gazave et al., 2009). In mammals, the key components of Notch signaling pathway are four Notch receptors, five Notch ligands and DNA-binding protein CSL [CBF-1/RBP-Jκ, Su(H), Lag-1]. Compared with other cell signaling pathways, Notch is relatively simple in structure and there is no second messenger involved in the activation process, so it cannot produce cascade amplification like others. The activation process of Notch signal pathway is as follows.

In the canonical Notch pathway, Notch receptors undergo two successive proteolytic cleavages (Bray, 2006; Kopan and Ilagan, 2009). After reaching the membrane and activated by the ligand on the neighboring cell, the Notch receptors can be cleaved by a disintegrin and metalloprotease (ADAM) family at Site 2 (S2) and then by γ-secretase at Site 3 (S3). Afterwards, Notch intracellular domain (NICD) was released to nucleus (Brou et al., 2000; Sprinzak et al., 2010). NICD translocates to the nucleus and forms a complex with the DNA-binding protein CSL and the coactivator Master-mind-like (MAML) family to regulate transcription of downstream target genes (Wu et al., 2000). In this way, the travelling NICD transduces the signal not only from cell to cell but also from extracellular to intracellular.

In the non-canonical Notch signaling mechanisms, the discovered interactions mainly focused on interplay between NICD and downstream effectors. For instance, NICD can directly interact with β-catenin (Jin et al., 2009), Smad proteins (Blokzijl et al., 2003), and HIF-1α (Gustafsson et al., 2005), thereby providing a crosstalk between Notch and the Wnt, TGFβ and hypoxia-dependent signaling pathways. However, it is worth mentioning that most Notch-correlated cancer phenotypes can be perturbed by the canonical rather than non-canonical Notch signaling.

Notch signaling regulates numerous cellular processes including cancer stem cell renewal, angiogenesis, proliferation, apoptosis, and EMT (Miele et al., 2006). More recently, it was reported that dysregulation of Notch signaling pathway was involved in endocrine resistance and combined Notch with estrogen signaling inhibition had showed synergistic effect in ERα positive breast cancer (Acar et al., 2016). In next section, we'll describe the potential mechanisms whereby Notch promotes endocrine resistance in depth.

## Notch Signaling and Endocrine Resistance in Breast Cancer

More freshly, Notch signaling was found to be an important pathway mediating endocrine resistance in breast cancer cells (Magnani et al., 2013). As an illustration, Paola Rizzo and his colleagues reported that Notch inhibition potentiated the effects of tamoxifen in ERα positive cells, T47D:A18. When they combined γ-Secretase inhibitors (GSI) and 4-OH-tamoxifen (Tam) together, combination treatment reduced the growth significantly more than either drug alone (Rizzo et al., 2008). These data suggested that GSIs may be a promising therapeutic target to overcome resistance for antiestrogen treatment. Besides Notch1, Yun et al. revealed that Notch4 also played an essential role in endocrine resistance as measurements of DNA content verified that Notch4-ICD in T47D:A18 increased DNA synthesis in the absence of estrogen, indicating that overexpression of Notch4-ICD could stimulate proliferation through estrogen-independent and Tam-resistant mechanisms (Yun et al., 2013).

To design new therapeutic strategies based on Notch signaling, Notch regulation and the context-dependent interactions between Notch and other relevant pathways needs to be taken into well consideration.

### The Regulation of ER by Notch Signaling

**Figure 1:**
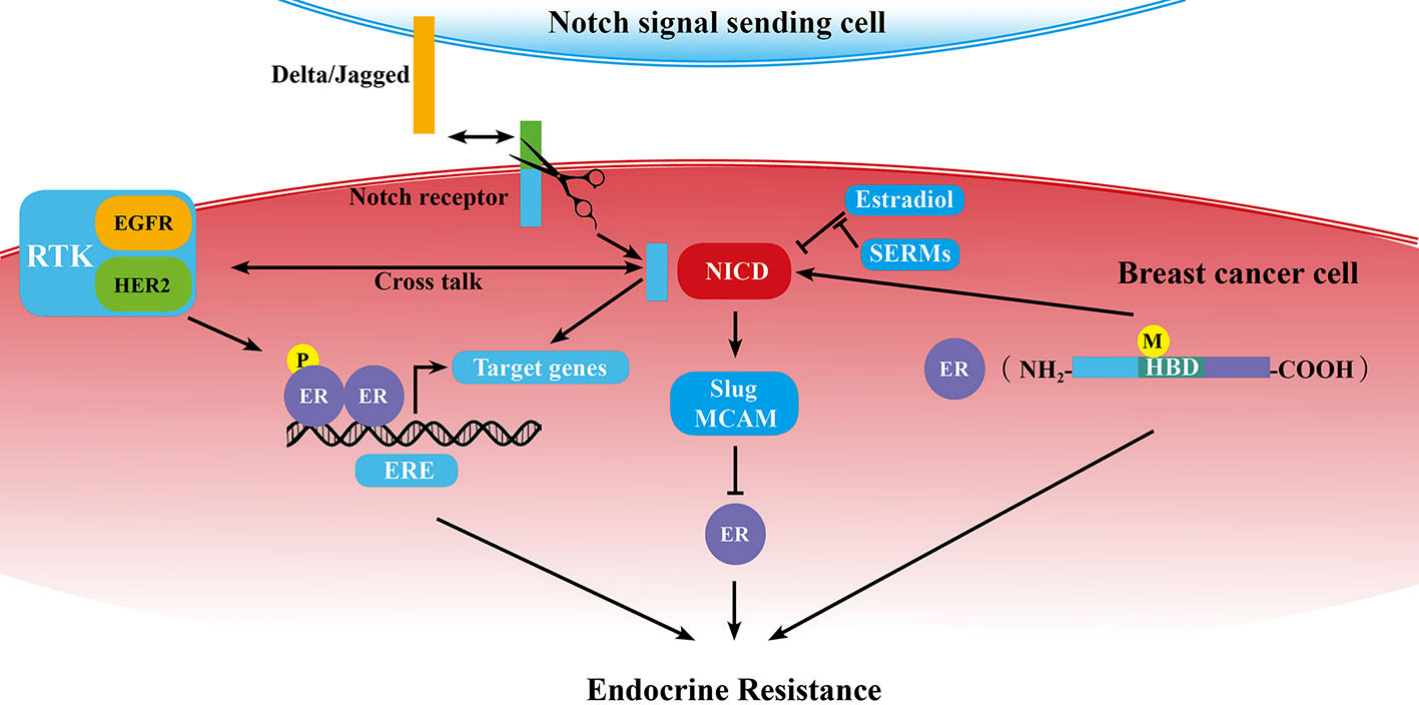
Schematic diagram of the correlation of Notch signaling pathway and ER in breast cancer endocrine resistance. ER is at the center of endocrine resistance observed in breast cancer cells. Notch signaling modulates endocrine therapy through cooperating with ER in a complex network as mentioned in some sections of this review. P, phosphorylation; M, mutation; →, promotion; ⊥, inhibition; ↔, receptor ligand-binding.

As we know, ERα dysregulation performs a central role in the acquisition of resistance to endocrine therapy in breast cancer. Previous studies demonstrated that Notch signaling pathway could directly or in-directly regulate ER expression or activity (**Figure 1**).

#### Regulation of ER Expression and Its Downstream Genes by Notch Family Members

There was interaction between the estrogen receptor and Notch in breast cancer (Rizzo et al., 2008). Breast cancer cells expressed Notch1 and Notch4 proteins at variable steady-state levels regardless of the ER status. But when examined basal centromere binding factor (CBF-1)–dependent reporter activity, researchers found an inverse correlation between Notch activity and ERα expression in breast cancer cells. In another word, activity of Notch was higher in MDA-MB231 cell line than in MCF-7 or T47D cell line. Notch activity was inhibited by estradiol (E2) significantly (*P* = 0.0025) *via* changing the cellular distribution of Notch1 in ERα positive (ERα+) cell lines and SERMs blocked its effect. In T47D:A18 cells (ERα + cell lines), GSI with an IC50 of 0.84 μmol/L exhibited strong inhibition effect on the growth *in vitro*. Moreover, combination of GSI and 4-OH-Tam had significantly more growth inhibition than either drug alone, even at very low concentrations. *In vivo*, treatment with GSI alone or tamoxifen alone blocked the growth of T47D:A18 xenografts with similar efficacy, but combination showed stronger effect. Another research (Hao et al., 2010) reported that E2 target genes, such as pS2, vascular endothelial growth factor-α (VEGFα), cyclin-D1, CD44, and c-Myc were upregulated by overexpression Notch1-ICD in the absence of E2. They further sought to identify the molecular mechanisms whereby Notch activated ER-dependent transcription without E2 using pS2 transcriptional model. They found Jagged-1 stimulated the recruitment of Notch1, IKKα, MAML1, p300/CBP, and ERα to the pS2 promoter by Chromatin immunoprecipitation assay (CHIP). The formation of above supramolecular complex contributed to activate a subset of ERα-responsive genes transcription in the absence of E2. In general, E2 inhibits Notch activation and SERMs reactivates Notch in breast cancer cells. Meanwhile Notch activates ERα-dependent transcription, demonstrating there is a feedback mechanism regulating the Notch1-ERα crosstalk. Altogether, Notch may promote endocrine resistance by affecting ERα activity.

Beyond that, some transcriptional factors, such as Snail (Scherbakov et al., 2012), Slug (Li et al., 2015; Bai et al., 2017), Twist (Vesuna et al., 2012), ZEB1 (Zhang et al., 2017), MCAM (Liang et al., 2017), were shown to mediate endocrine resistance through directly repressing ERα expression. In breast cancer cells, Notch1 (Shao et al., 2015) or Notch4 (Zhou et al., 2020) can promote the expression of Slug by activating the Slug promoter. We recently published an article which showed Notch1 could also transcriptionally activate MCAM in breast cancer cells (Zeng et al., 2020). So Notch1-MCAM signaling pathway is possibly another method leading to endocrine resistance in breast cancer. In a word, apart from directly activation of ERα downstream gene expression, Notch1/4 may indirectly influence ERα expression contributing to endocrine resistance.

In contrast to Notch1 and 4, our previous study (Dou et al., 2017) showed that Notch3 was mainly expressed in luminal breast cancer cells but not in either basal-like or HER2 (human epidermal growth factor receptor 2)-positive breast cancer cell line. Notch3 expression displayed strong positive correlation with ERα both in protein and mRNA level. When Notch3 was silenced *via* siRNA, ERα was decreased. Conversely, overexpression of Notch3 resulted in upregulation of ERα. We also found that Notch3 specifically bound to the CSL binding element of the ERα promoter and transcriptionally activated ERα expression by CHIP and Electrophoretic mobility shift assay (EMSA). In addition to such direct regulation, it was also found Notch3 could indirectly increase ERα expression by GATA3. We found that protein and mRNA level of Notch3 and GATA-3 was positively correlated especially in luminal breast cancer cells. There were two putative CSL-binding sites located upstream of GATA-3 promoter (-829-834 bp and -665-670 bp). CHIP, EMSA, and dual reporter assay certificated Notch3 activated GATA-3 transcription by binding to CSL-binding elements in the GATA-3 promoter in MCF-7 and MDA-MB231 cells (Lin et al., 2018). GATA3 helped to maintain a luminal phenotype by activating ERα (Eeckhoute et al., 2007). Unlike Notch1 and Notch4, the role of Notch3 in anti-estrogen therapy need to further research, perhaps Notch3 expression can increase sensitivity to endocrine therapy.

#### ER Mutations, ER Modification, and Notch Signaling

A recent study confirmed that hot spot mutations in hormone binding domain (HBD) of ERα/ESR1 like Y537N, Y537S, D538G alterations, promoted transcription in an ER-dependent manner and proliferation even in the absence of estrogen, leading to endocrine resistance (Toy et al., 2013). Compared with wild-type ESR1, mutant cells displayed an increase in CD44^+^/CD24^-^ ratio, mammosphere formation, migratory capabilities, and self-renewal through highly expressed Notch signaling components, like receptors, ligands and target genes (Gelsomino et al., 2018). It also demonstrated that ERα-Y537S could not enhance BCSCs once Notch signaling was inhibited, which reaffirming the importance of the correlation between ER and Notch in ERα-Y537S-mediated BCSC enrichment. Therefore, the development of Notch inhibitors will be new strategies to prevent or delaying disease progress and relapsing-onset in ER mutant positive patients.

Apart from the above mutations, modification of ERα such as phosphorylation, contributing to ligand-independent transcription of ERα-dependent genes, also promoted resistance to anti-estrogen therapy (Korobeynikov et al., 2019). HBD-ERα mutants exhibited an overexpressed S118 phosphorylation located within the AF-1 domain. When transfecting S118A-ERα (a plasmid where a serine was changed to an alanine to eliminate phosphorylation at S118) in MCF-7 cell, it was detected a lower expression of Notch4-ICD, Notch4 and HES1, and reduction in mammosphere forming efficiency (MFE) only in Y537S-ERα mutant cells. These results indicated that Notch4 activation was required for phosphorylation of S118 to increase BCSC activity in ERα mutant cells (Gelsomino et al., 2018). In addition, it was reported that receptor tyrosine kinases (RTK) and several other pathways, including the CDK2 complex and CDK7/TFIIH complex enhanced the phosphorylation of ERα (Rani et al., 2019). RTK includes epidermal growth factor receptors (EGFR), vascular endothelial growth factor receptor (VEGFR), insulin-like growth factor-I receptor, et al. RTKs promoted ER phosphorylation through at least two pathways: RAS–RAF–ERK and PI3K-AKT pathway (Ali and Coombes, 2002), which enabled ERα positive breast cancer cells to escape from anti-estrogen therapies. Furthermore, a meaningful crosstalk existed between Notch and the RTK. When overexpressing active Notch1, EGFR expression was increased. On the other hand, Notch1 overexpression could reverse EGFR inhibitor-induced cell toxicity, suggesting mutual positive regulation existed between Notch1 and EGFR (Dai et al., 2009). In addition, a study which analyzed the statistical data of histological and immunophenotypic parameters from 98 invasive breast cancer patients found that Notch2 and HER2, also known as human epidermal growth factor receptor 2, had positive correlation (Florena et al., 2007). In this way, Notch may indirectly promote endocrine resistance by ER phosphorylation through RTK pathways.

In brief, HBD-ESR1 mutations and ERα phosphorylation result in endocrine resistance and subsequent progression or relapse by means of increased BCSC activity induced by activating Notch signaling. Though the early detection of ER mutations is an immense difficulty for breast cancer, developing approaches targeting Notch pathway to prevent disease development and metastatic will be a valuable clinical decision.

### The Crosstalk of Notch and Other Signaling Pathway Which Involves in Endocrine Resistance

In addition to the direct regulation of ER expression, Notch could cooperate with other pathways and cause endocrine resistance.

There is an abundance of evidence that the number of breast cancer stem cells (BCSCs) rose during antiestrogen treatment for ERα positive tumors (Creighton et al., 2009; O'Brien et al., 2011). Notch1 and Notch4 have been validated to regulate breast cancer stem cells by recent studies (Harrison et al., 2010; Gonzalez et al., 2014). Harrison H and his colleagues demonstrated that in stem cell-enriched cell populations, Notch4 signaling activity was elevated to 8-fold than differentiated cells; however, Notch1 signaling was only 4-fold higher. Their finding verified that Notch4 may produce more robust effect in maintaining breast cancer stemness (Harrison et al., 2010). Simões BM declared that short-term treatment with antiestrogens agents impaired cell proliferation yet improved breast CSCs activity through Jagged-1/Notch4 receptor activation in tumor tissue derived from breast cancer patients and xenograft (PDX) tumors (Simoes et al., 2015). Another study also showed that in ER positive breast cancer treatment with FKBPL-based therapeutics inhibited endocrine therapy resistant stem cells *via* downregulating DLL4 and Notch4 (McClements et al., 2019). In breast cancer, from non-CSCs to CSCs, CSC activity could be stimulated following exposure to estrogen *via* paracrine signaling. *In vitro* and *in vivo*, Gefitinib (EGFR inhibitor) and GSI were proven to barricade CSC activity induced by estrogen and GSIs showed more effective than Gefitinib (Harrison et al., 2013). In sum, these evidences demonstrated that detected Notch-sensitive CSCs might predict endocrine sensitivity and using Notch blockade may be an effective therapeutics for breast cancer.

It has been claimed that Notch signaling plays critical roles in acceleration of EMT in breast cancer cells which are drug-resistant. Results from Bui QT and his colleagues revealed that mesenchymal marker proteins in Tam-resistant human breast cancer (TamR-MCF-7) cells were highly expressed compared to MCF-7 cells. They proved Notch4 was instrumental in regulating EMT signaling in TamR-MCF-7 cells, but not Notch1. These results might hit upon a potential strategy to prevent metastasis in TAM-resistant breast cancer (Bui et al., 2017). Lombardo Y also found that endocrine therapies resistant cells overexpressed Nicastrin and Notch4 with mesenchymal phenotype (Lombardo et al., 2014). In another paper, it was declared that DMXL2 was increased in some endocrine therapy resistant breast cancer cells where DMXL2 promoted EMT *via* activating Notch signaling through V-type ATPase dependent acidification (Faronato et al., 2015).

Recent evidence displayed that Notch signaling pathway was engaged in the differentiation of tumor-associated macrophages (TAMs) in breast cancer (Palaga et al., 2018). Liu H detected that increased upregulation levels of Jagged-1 led to macrophage differentiation toward M2-TAMs (Liu et al., 2017). TAM secreted CC-chemokine ligand 2 (CCL2), which resulted in breast cancer endocrine resistance by activation of the PI3K/Akt/mTOR pathway (Li et al., 2020). Adipocytes and breast cancer cells could secrete Interleukin (IL) 6. IL-6 was found to trigger a potential Notch3/Jagged-1 loop in autocrine/paracrine mode to boost BSCS self-renewal in the mammary gland (Sansone et al., 2007). Sansone P revealed that the inhibition of IL6R/IL6-Notch3 pathways combined with hormone therapy restored ERα expression and switched CD133^hi^ self-renewal from IL6/Notch3-dependent to an ER-dependent one (Sansone et al., 2016).

Moreover, Rizzo and his colleagues demonstrated that no matter in MDA-MB231 or T47D cell, Notch1 knockdown or GSI treatments led to cyclins A and B1 downregulation, thus G2 arrest. In T47D cells, Notch inhibition strengthened the effects of tamoxifen. And *in vivo*, GSI, and tamoxifen treatment caused regression of T47D tumors (Rizzo et al., 2008). To this extent, Notch is capable of promoting endocrine resistance by regulate cell cycle.

## Conclusions

Hormone receptor positive breast cancer accounts for 70% of all breast cancer patients. For this type of patients, despite advances in therapy, antiestrogen drugs to block ERα function is still the most meaningful approach. Unfortunately, a considerable proportion of tumors eventually develop resistance during the course of the treatment. Therefore, there is an urgent requirement to study the underlying resistance mechanism and identify novel targets in hormone receptor positive breast cancers for therapeutic intervention. Lately, increasing preclinical and clinical evidence had shown that Notch signaling pathway led to antiestrogen resistance which was related with the regulation of ER expression/activity, maintenance of CSCs and mesenchymal phenotype, crosstalk with other tyrosine kinase growth factor signaling pathways and impact on tumor microenvironment. Takebe et al. demonstrated that in Phase II clinical trials, therapy of GSI MK-0752 combined with docetaxel effectively improved the health of patients who had advanced breast cancer, indicating that chemotherapy resistance might be reversed by targeted inhibition of Notch pathway (Takebe et al., 2014). But no clinical results are launched about combination of GSI with antiestrogen. Treatment targeting both ER and Notch may hold a promising future in overcoming endocrine resistance.

## Author Contributions

J-WB and MW designed the project and wrote the manuscript (they contributed equally to this work). J-WL searched related articles from PubMed, Medline, and Google Scholar. The whole project was arranged and supervised by G-JZ. All authors contributed to the article and approved the submitted version.

## Funding

This work was supported in part by grants from National Natural Science Foundation of China (No.81602345, 91859120), Research Team Project of Natural Science Foundation of Guangdong Province (2016A030312008) Science and Technology Planning Project of Xiamen (3502Z20194040), Xiamen's Key Laboratory of Precision Medicine for Endocrine-Related Cancers, and start-up fund from Xiamen University.

## Conflict of Interest

The authors declare that the research was conducted in the absence of any commercial or financial relationships that could be construed as a potential conflict of interest.
